# Association between hyperglycemia on admission and preoperative deep venous thrombosis in patients with femoral neck fractures

**DOI:** 10.1186/s12891-022-05862-0

**Published:** 2022-10-06

**Authors:** Wei Yao, Wanyun Tang, Wei Wang, Qiaomei Lv, Wenbo Ding

**Affiliations:** 1 Department of Orthopedics, Dandong Central Hospital, China Medical University, No. 338 Jinshan Street, Zhenxing District, Dandong, Liaoning Province 118002 P.R. China; 2 Department of Oncology, Dandong Central Hospital, China Medical University, No. 338 Jinshan Street, Zhenxing District, Dandong, Liaoning Province 118002 P.R. China

**Keywords:** Femoral neck fracture, Deep venous thrombosis, Risk factor, Hyperglycemia

## Abstract

**Background:**

Elevated blood glucose is the most frequent electrolyte disturbance in acutely ill patients. This study aimed to determine whether admission hyperglycemia is associated with the incidence of preoperative deep venous thrombosis (DVT) in patients with femoral neck fractures.

**Methods:**

This retrospective study was conducted on consecutive patients with femoral neck fractures admitted to our institution from March 2018 to March 2022. Blood glucose levels were measured within 24 h of admission and categorized into quartiles (Q1 = 5.30; Q2 = 5.70; Q3 = 6.60). Patients were divided into four groups (Group1-4) based on the quartiles. Preoperative DVT was diagnosed using venous compression ultrasonography. Multivariable logistic regression models and propensity score matching analysis evaluated the association between blood glucose and preoperative DVT in patients.

**Results:**

Of 217 patients included in this study, 21(9.7%) had preoperative DVT in hospital, and admission hyperglycemia was observed in 83 (38.2%). Preoperative DVT was higher in patients with hyperglycemia (*n* = 15) than patients without hyperglycemia (*n* = 6) in the multivariable logistic regression models (OR 3.03, 95% CI 0.77–11.87). Propensity scores matching analyses manifested that compared with patients with group 2 (5.30 – 5.70 mmol/L) of glucose levels, the odds of preoperative DVT were slightly higher (OR 1.94, 95% CI 0.31–12.12) in patients with group 3 (5.70 – 6.60 mmol/L), substantially higher (OR 6.89, 95% CI 1.42–33.44, P trend < 0.01) in patients with the group 4 (> 6.60 mmol/L) of glucose levels.

**Conclusions:**

In patients hospitalized for femoral neck fracture, markedly elevated blood glucose is associated with increased preoperative DVT in patients. The development of this biomarker could help in guiding patient counseling, risk assessment, and future management decisions.

**Supplementary Information:**

The online version contains supplementary material available at 10.1186/s12891-022-05862-0.

## Introduction

Femoral neck fractures are a disease with high morbidity and mortality, caused by various factors [1, 2]. In elderly people, femoral neck fractures are commonly caused by osteoporosis and low-energy injury [3]. In contrast, high-energy femoral neck fractures mainly occur in young and middle-aged people, mostly caused by falls from heights and various car accidents [4]. The number of femoral neck fractures is projected to increase dramatically to 4.5 million by 2050 due to the accelerating aging of the global population [5]. Currently, femoral neck fractures are primarily treated with surgery [6]. For elderly femoral neck fractures, non-displaced fractures can be treated with conservative or internal fixation (IF), while displaced fractures are mostly treated with arthroplasty. For young femoral neck fractures caused by high energy, internal fixation (IF) should be performed early to make the fracture heal [7].

Although the diagnosis and treatment of femoral neck fracture have greatly improved in recent years, the cannulated screw system (CSS) is still one of the most commonly used internal fixation methods. However, the overall treatment effect remains flawed due to its associated adverse factors (operative failure rate, femoral head necrosis rate and fracture nonunion rate) [8, 9]. Therefore, it is emphasized that early surgery for femoral neck fractures, anatomical reduction as much as possible, and perioperative application of an orthopedic-led interdisciplinary model of care may reduce the occurrence of adverse risk factors [10]. Meanwhile, further research is needed to accurately assess prognostic factors and predictive models in patients with femoral neck fractures to improve clinical outcomes.

Risk factors generally considered to contribute to poor prognosis of femoral neck fractures include deep vein thromboembolism, advanced age, cancer, stroke, venous insufficiency, obesity, pregnancy, trauma, hypertension, history of diabetes, etc. [6, 11, 12]. Among them, deep vein thrombosis (DVT) is the most frequent complication influencing poor prognosis in patients with femoral neck fractures.

Deep vein thrombosis (DVT) is abnormal clotting of blood in deep veins that can lead to pulmonary thromboembolism (PTE), which can lead to sudden death [13]. According to reports, 75–269 cases of venous thromboembolism (VTE) per 100,000 individuals in Europe, America, Australia, and Southern Latin America, and PTE accounts for one-third of all VTE cases [14]. As the most common complication in patients with femoral neck fractures, DVT not only increases the medical burden on society but also threatens the patient's life [15, 16]. Therefore, understanding the epidemiological characteristics of DVT and preventing the occurrence of DVT is the focus of clinical work.

Admission hyperglycemia is common after femoral neck fractures in the acute phase, even in nondiabetic patients [17]. Femoral neck fractures induce a state of systemic stress, stimulating the release of glucocorticoids and reducing insulin sensitivity, thereby resulting in acute hyperglycemia [18]. In recent years, acute hyperglycemia has been reported in several thrombotic conditions, including myocardial infarction (MI), stroke, and venous thromboembolism (VTE) [19]. Amit Akirov et al. showed that increased coagulation factors and impaired fibrinolysis are associated with acute hyperglycemia [20]. Moreover, acute hyperglycemia may influence clinical outcomes even without diabetes [21]. Since VTE is a serious complication of fractures, several studies have investigated the relationship between VTE and hyperglycemia. Marjolein K. Sechterberge et al. found that hyperglycemia after hip surgery is associated with activation of coagulation and increased risk of venous thromboembolism [22]. Hermanides j et al. described that during hip surgery, blood glucose in patients without diabetes increased, and coagulation was activated [23]. An increasing body of evidence shows that acute hyperglycemia is closely related to the occurrence of thrombotic events and mortality in patients with hip fractures after surgery [22–25]. However, there was limited information between admission hyperglycemia and preoperative DVT in patients with femoral neck fractures waiting for surgery [26].

Based on the hypothesis that admission hyperglycemia is related to preoperative thrombotic events, this study aims to analyze the association between hyperglycemia on admission and preoperative DVT following femoral neck fractures in our institution of patients whose preoperative DVT was derived from highly accurate and complete databases of the ultrasonic detection system.

## Methods

### Study design

In this retrospective, single-center cohort study, data on patients with femoral neck fractures were collected from the consecutively electronic health record of Dandong central hospital, China medical university, from March 2018 to March 2022.

An institutional review board of Dandong central hospital's ethics committee approved this study. A waiver of consent was sought and obtained for this cohort study by the ethics committee.

### Patient selection

Inclusion criteria were as follows (1) Age ≥ 18 years, (2) Fresh femoral neck fractures that require treatment in our hospital, (3) Patients with femoral neck fractures were evaluated based on imaging (including X-ray, CT, or MRI), physical examination, or intraoperatively by an orthopedist, (4) Informed consent form signed and willing to undergo anticoagulant therapy for preventing thrombosis, (5) Results of preoperative ultrasound are available, (6) A total blood glucose measurement obtained on admission.

Exclusion criteria were as follows (1) Repeat admissions or no documentation of any DVT examination, (2) Multiple fractures, (3) Past peripheral vascular disease, recent anticoagulant therapy, (4) The length of stay was less than three days, (5) DVT was diagnosed before the injury.

### Blood glucose measurement

Admission blood glucose was defined as the first (non-fasting) venous blood glucose measured in the acute phase (within 24 h after admission) after femoral neck fractures. We arranged all the values of the patient's admission blood glucose from small to large and categorized them into quartiles, which were expressed by Q1 (= 5.30), Q2 (= 5.70) and Q3 (= 6.60), respectively. Patients were divided into four groups (Group1-4) based on the quartiles. For group 1 (lowest-Q1) through 4 (highest-Q3), blood glucose levels were grouped by quartile using cut points of 0.00–5.30, 5.30- 5.70, 5.70–6.60, and > 6.60 mmol/L, respectively. By the 2020 American Diabetes Association guidelines, a standard glycemic control goal is less than 10.00 mmol/L, while tight glycemic control goal is 6.10–7.80 mmol/L [27]. Thus, We also defined normoglycemia and hyperglycemia as blood glucose 4.00–6.09 mmol/L and > 6.10 mmol/L.

### Data collection

We collected the details (age and sex) of patients and acquired the history of smoking and drinking. As well as the history of the disease process (including Hypertension, Diabetes, Coronary artery disease, Cerebrovascular disease, Chronic renal failure, Chronic pulmonary disease, and Malignancy). The time from hospitalization to operation and injury to admission were used to assess fracture status. Fractured limbs categorized patients on admission. Family history of VTE and some baseline biomarker concentrations (including White blood cell count, Neutrophil count, Lymphocyte count, Platelet count and D-Dimer count) were also collected.

### Outcome measure

The primary outcome was preoperative deep venous thrombosis. Preoperative DVTs were classified into two types: proximal and distal. An iliac and femoral vein thrombus was considered proximal thrombosis, and a distal thrombus was considered thrombosis in calf veins. We routinely performed bilateral Doppler ultrasonography on each included patient after admission. Radiologists diagnosed preoperatively DVT based on Harish Patel's report [28] and reviewed by a senior radiologist. ACCP guidelines (2016, 10th edition) were followed if a preoperative DVT was identified. According to the guidelines [29], we used low molecular weight heparin (LMWH) as the primary prophylaxis. Rivaroxaban was used as an alternative in patients who refused to inject LMWH.

### Statistical analysis

For baseline cohort characteristics, the means (with standard deviation) of continuous variables were reported and compared using analysis of variance. Categorical variables were expressed as counts (frequency distributions) and compared using the χ2 test. *P* values less than 0.05 (2-sided) were considered significant in all trials of significance, regardless of the number of sides. All variables were imputed with multiple imputations because, compared to other methods of handling missing data, this method seemed less biased.

Multivariable logistic regression models were used to adjust traditional risk factors. Data were presented with Odd ratio (OR) and 95% CI. Using univariate analyses, characteristics derived from a value of *p* < 0.10 were integrated into multivariate logistic regression models.

A propensity score-matched analysis was used to minimize bias from confounding variables. We used a propensity score-matched sample to minimize pre-existing imbalances in selected covariates between the hyperglycemia and normoglycemia groups. According to previous studies [17–19, 25, 30], these selected covariates mainly included age, sex, smoking, alcohol abuse, time from hospitalization to operation, family history of VTE, hypertension, diabetes, coronary artery disease, cerebrovascular disease, chronic renal failure, chronic pulmonary disease, time from injury to admission, fractured limbs and other essential biomarkers (White blood cell, Neutrophils, Lymphocyte, Platelet, and D-Dimer). Propensity score matching of 2 similar groups was conducted with 1:1 ratio and a match tolerance of 0.25SD. We used absolute standardized differences to compare the characteristics of hyperglycemic and normoglycemic patients after 1:1 propensity score matching; a difference more than 0.1 is considered meaningful.

All statistical analyses were conducted using SPSS version 26 (SPSS Inc) and R software version 4.0.3 (Matching and Frailty Pack packages, R Foundation for Statistical Computing).

## Results

A total of 217 patients with femoral neck fractures fulfilled our criteria (eFigure 1). Among them, 21 (9.7%) patients with preoperative DVT, admission hyperglycemia was observed in 83 (38.2%) patients and normal glycemia in 134 (61.8%) patients. Table 1 presents the baseline characteristics of patients by severity of blood glucose. Participants with admission hyperglycemia were more likely to be presented with hypertension and diabetes, easier to have a family history of VTE and cerebrovascular disease, and higher white blood cell count.

**Table 1:** Baseline characteristics of the patients by blood glucose (mmol/L)

Characteristics	Total patients (*n* = 217)	Blood glucose Quartile(mmol/L)				P for Trend†
		Group 1 0.00–5.30 (*n* = 67)	Group 2 5.30–5.70 (*n* = 49)	Group 3 5.70–6.60 (*n* = 47)	Group 4 > 6.60 (*n* = 54)	
Demographics						
Mean age, years (SD)	73(11.2)	72.2(12.5)	72.8(12.7)	74.5(10.0)	73.0(9.1)	0.58
Female gender	152(70.0)	43(64.2)	37(75.5)	32(68.1)	40(74.1)	0.51
Current Smoking	41(18.9)	16(23.9)	6(12.2)	9(19.1)	10(18.5)	0.47
Alcohol abuse	42(19.4)	14(20.9)	7(14.3)	10(21.3)	11(20.4)	0.79
Meantime from hospitalization to operation, days (SD)	5.5(3.2)	5.2(3.3)	5.4(2.7)	5.4(3.2)	6.1(3.6)	0.44
Family history of VTE	46(21.2)	9(13.4)	11(22.4)	7(14.9)	19(35.2)	0.02
Comorbidity						
Hypertension	102(47.0)	23(34.3)	21(42.9)	23(48.9)	35(64.8)	0.01
Diabetes	44(20.3)	5(7.5)	2(4.1)	5(10.6)	32(59.3)	< 0.001
Coronary artery disease (CAD)	33(15.2)	14(20.9)	10(20.4)	4(8.5)	5(9.3)	0.12
Cerebrovascular disease	60(27.6)	11(16.4)	17(34.7)	11(23.4)	21(38.9)	0.03
Chronic renal failure	17(7.8)	7(10.4)	3(6.1)	3(6.4)	4(7.4)	0.81
Chronic pulmonary disease	22(10.1)	10(14.9)	3(6.1)	6(12.8)	3(5.6)	0.25
Malignancy	12(5.5)	2(3.0)	1(2.0)	5(10.6)	4(7.4)	0.20
Time from injury to admission						
≤ 12 h	105(48.4)	23(34.3)	28(57.1)	24(51.1)	30(55.6)	0.12
12–24 h	44(20.3)	15(22.4)	10(20.4)	11(23.4)	8(14.8)	
≥ 24 h	68(31.3)	29(43.3)	11(22.4)	12(25.5)	16(29.6)	
Fractured limbs						
Left	112(51.6)	36(53.7)	28(57.1)	22(46.8)	26(48.1)	0.70
Right	105(48.4)	31(46.3)	21(42.9)	25(53.2)	28(51.9)	
Baseline biomarker concentrations (Mean, SD)						
White blood cell count, × 10^9/L	8.4(2.4)	7.5(2.2)	8.2(2.5)	8.8(2.1)	9.4(2.4)	0.002
Neutrophil count, × 10^9/L	6.3(2.3)	5.4(2.0)	6.1(2.4)	6.6(2.3)	7.3(2.3)	0.15
Lymphocyte count, × 10^9/L	1.3(0.6)	1.4(0.6)	1.3(0.5)	1.3(0.4)	1.4(0.7)	0.95
Platelet count, × 10^9/L	221.3(66.0)	207.2(69.6)	207.3(69.7)	226.3(65.4)	207.1(58.0)	0.50
D-Dimer count, × ug/ml	6.4(6.5)	4.9(5.8)	7.0(6.2)	6.4(6.6)	7.7(7.2)	0.39

^†^ *P* values for linear trend for continuous variables are from a generalized linear model, and categorical variables are from an ordinal or logistic regression

A comparison of patient demographics with preoperative deep venous thrombosis stratified by normal glycemia and hyperglycemia was shown in Table 2. The statistical analysis produced 83 patients with hyperglycemia; among them, 15 (18.1%) patients with preoperative DVT, 7 (8.4%) patients with proximal DVT, and 8 (9.6%) patients with distal DVT. Hyperglycemia was associated with higher DVT incidence.

**Table 2:** Comparison of deep venous thrombosis (DVT) by Glucose Level (≥ 6.10 mmol/L and < 6.10 mmol/L) (*n* = 217)

Type of venous thromboembolism	No. (%)		*p*-value
	Blood glucose < 6.10 mmol/L (*n* = 134)	Blood glucose ≥ 6.10 mmol/L (*n* = 83)	
DVT	6(4.5)	15(18.1)	0.001
Proximal	2(1.5)	7(8.4)	0.01
Distal	4(3.0)	8(9.6)	0.04

**Fig. 1:**
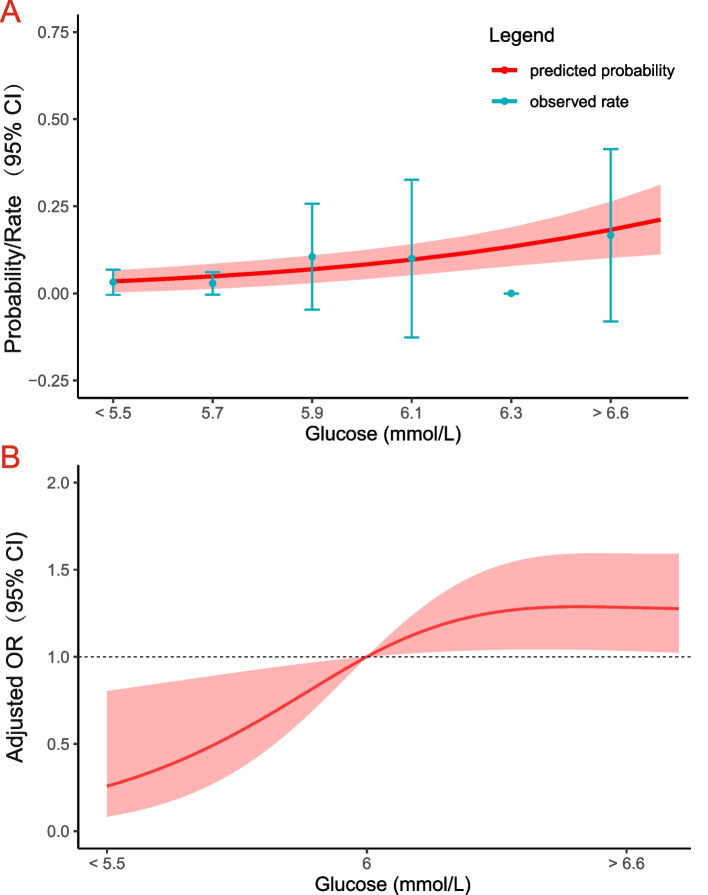
Relationship between baseline blood glucose level and DVT in patients with femoral neck fractures. **A** Predicted probabilities and the observed rate of DVT. **B** Adjusted odds ratio (OR) and 95% confidence interval (CI) are shown for each 0.5 mmol/L change away from the reference value (6 mmol/L). DVT: deep venous thrombosis. *Blood glucose Levels were logarithmically transformed, and a multivariate model was adjusted for smoking, time from hospitalization to operation, family history of VTE, Hypertension, Diabetes, cerebrovascular disease, d-dimer count

The dose–response relationship between blood glucose and preoperative DVT was also shown in Fig. 1. Based on baseline blood glucose levels, predicted probabilities and observed rates of DVT were increased. Further, patients with higher blood glucose had a higher risk of preoperative DVT compared to those with blood glucose less than 5.5 mmol/L (reference).

After adjusted for all covariates in multivariable regression analysis (Table 3), the frequency of preoperative DVT was significantly higher in hyperglycemic patients than in normoglycemic patients (OR 3.03, 95% CI 0.77–11.87). Preoperative DVT was still significant in propensity score matching analysis (OR 2.52, 95% CI 0.62–10.28).

**Table 3:** Comparison of the unadjusted and risk-adjusted outcomes by glucose level (≥ 6.10 mmol/L and < 6.10 mmol/L)

Outcome	Unadjusted		Multivariable Regression Adjustment		Propensity Score Adjustment	
	OR (95% CI)	p	OR (95% CI)	p	OR (95% CI)	*p*-Value
Deep venous thrombosis	4.71(1.75–12.68)	0.002	3.03(0.77–11.87)	0.11	2.52(0.62–10.28)	0.20

Based on the quartiles of blood glucose levels (Group1-4), the patients were then divided into four groups for their outcomes of preoperative DVT (Table 4). Univariate analysis demonstrated that elevated blood glucose levels were significantly associated with preoperative DVT. Multivariable logistic regression analysis identified smoking, time from hospitalization to operation, family history of VTE, hypertension, diabetes, cerebrovascular disease, and d-dimer count as independent predictors of preoperative DVT (eTable1 in Data Supplement). Compared with patients with Group 2 (5.30–5.70 mmol/L), the odds of DVT were trending higher (but not significant) in patients with Group 3 (OR 1.21, 95% CI 0.20–7.40) and significantly higher in patients with Group 4 (OR 3.35, 95% CI 0.52–21.49). There was still an association between higher blood glucose levels and increased odds of DVT even when blood glucose levels were analyzed as a continuous variable. (See Fig. 1 for a dose–response plot). The trend remained significant among propensity score-matched groups (P trend < 0.01): compared with Group 2, the odds of DVT were slightly higher with Group 3 (OR 1.94, 95% CI 0.31–12.12), significantly higher with Group 4 (OR 6.89, 95% CI 1.42–33.44). The detailed variates matching between the two groups are in the eTable 2, 3, 4 and 5  in Data Supplement.

**Table 4:** Unadjusted and adjusted associations between quartile of blood glucose levels and deep venous thrombosis

Outcome	Quartile of Glucose (mmol/L)	Events, n (%)	Unadjusted OR	P trend 1	Multivariable Regression adjusted OR	P trend 2	PSM adjusted OR	P trend 3
Deep venous thrombosis	Group 1 [0.00–5.30]	0(0)	NA	< 0.001	NA	0.01	NA	0.004
	Group 2 [5.30–5.70]	4(8.2)	1 [Reference]		1 [Reference]		1 [Reference]	
	Group 3 [5.70–6.60]	4(8.5)	1.05(0.25–4.45)		1.21(0.20–7.40)		1.94 (0.31–12.12)	
	Group 4 [> 6.60]	13(24.1)	3.57(1.08–11.82)		3.35(0.52–21.49)		6.89(1.42–33.44)	

**Fig. 2:**
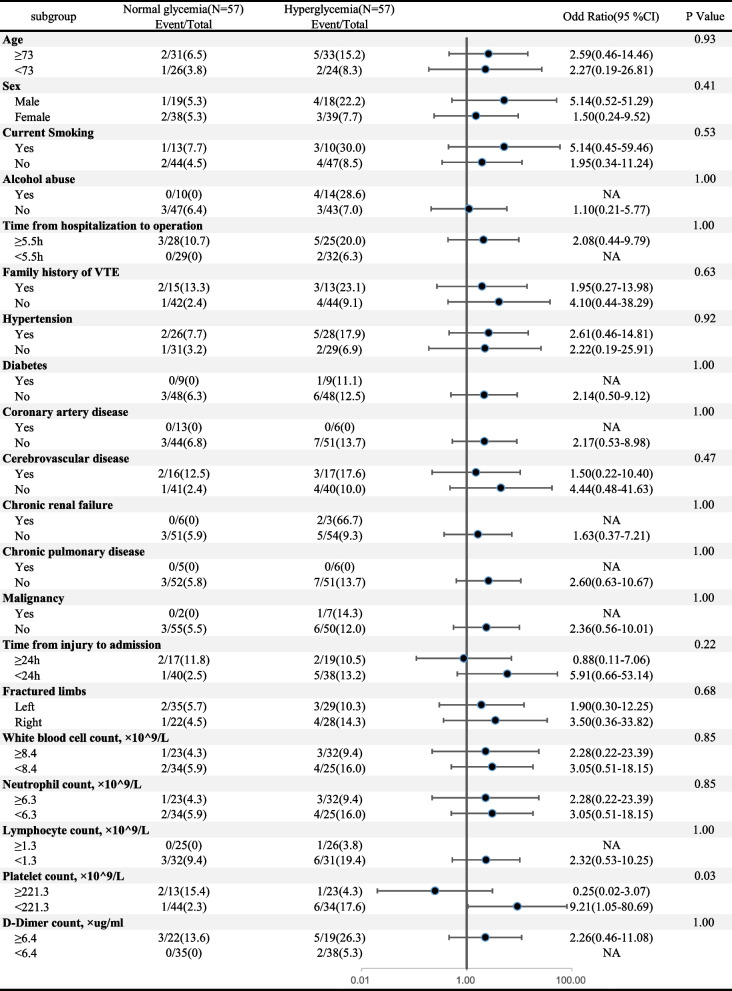
Subgroup Analysis of association between blood glucose levels and deep venous thrombosis after propensity score matching. *Multivariate model adjusted for smoking, time from hospitalization to the operation, family history of VTE, hypertension, diabetes, cerebrovascular disease, d-dimer count

In addition, we examined the interactions of factors on hyperglycemia (Fig. 2). Effect modification is observed with platelet count (P for interaction < 0.05). Other variables are not significantly affected by hyperglycemia and preoperative DVT.

## Discussion

This retrospective, single-center cohort study found that hyperglycemia was associated with an increased occurrence of preoperative DVT compared with normoglycemia. Further, a dose–response association was indicated between patients with hyperglycemia at admission and preoperative DVT.

To our knowledge, this is the first retrospective study focusing on the association between admission hyperglycemia and preoperative DVT in patients with femoral neck fractures. Most studies focused on evaluating the risk factors of deep vein thrombosis after hip fracture [1, 6, 11, 12, 31]. These studies suggested age, female, fibrinogen, movement disorder, bedridden time, diabetes, and d-dimer as risk factors for developing DVT after fractures. Moreover, some studies evaluated the risk factors of postoperative DVT in patients with femoral neck fractures [32, 33]. There was limited understanding of the risk factors of preoperative DVT after femoral neck fractures [34–36]. Other studies assessed the association between admission hyperglycemia and adverse events in patients with hip fractures and showed varied and conflicting results [25, 26, 30, 37]. The following study by John Thorling et al. found that elevated admission HbA1c was not associated with postoperative adverse outcomes in hip fracture patients [37]. By contrast, Riccardo Leto and Masayuki Iki et al. demonstrated that admission hyperglycemia was associated with increased complications in patients with hip fractures [26, 30]. Boris Mraovic et al. did find that increased glucose levels were associated with venous thrombosis [25]. Additionally, previous studies have defined thresholds based on two categories [36–38]. In this study, a dose–response relationship was found between glucose level and preoperative DVT.

The exact mechanism explaining such an association between hyperglycemia and preoperative DVT remains unclear. However, several previous studies might show a causal relationship between hyperglycemia and DVT [16, 22, 25]. According to Mraovic et al., following major orthopedic surgery, hyperglycemia significantly increases the risk of pulmonary embolism [25]. Hyperglycemia induces coagulation activation and downregulation of fibrinolytic activity due to increased levels of several procoagulant factors, including thrombin-antithrombin, as described by Marjolein K. Sechterberger et al. [22]. Further, hyperglycemia impairs innate immunity, resulting in systemic anti-inflammatory responses [16].

Interestingly, in the subgroup analysis, this study found that hyperglycemia was associated with preoperative DVT in patients with low platelet count (< 221.3 × 10^9/L) but not in those with high platelet count (≥ 221.3 × 10^9/L), with the assistance of a statistical test for interaction (*p* = 0.03). While the mechanism is ambiguous, it should be interpreted circumspectly since multiple subgroups can lead to spurious positive results [39].

Different from the previous studies, our study found DVT did not increase as a result of pulmonary disease, hypertension, or patient's gender [11]. In addition to being associated with hyperglycemia, preoperative DVT was also correlated with the d-dimer count and time from hospitalization to operation, which is significantly consistent with the previous studies [12, 15, 40]. For detecting acute DVT in non-traumatic environments, the D-dimer is a valuable modality that possesses a high sensitivity but low specificity [40]. Patients with long-time from hospitalization to operation might have a higher risk for DVT [12]. It means they may need close surveillance and surgery as soon as possible to avoid preoperative DVT.

This study has the following notable strengths: First, we used propensity score matching and multivariable logistic regression to adjust for confounders. Second, there appeared to be a readily discernible dose–response relationship. Third, our results remained remarkable after including most other important biomarkers, including white blood cells, neutrophils, lymphocytes, platelet, and d-dimer.

Nevertheless, this study has several limitations. Firstly, the preoperative DVT sample size was small. Secondly, blood glucose levels were not measured daily for all patients in this study. A dynamic monitoring system would be more valuable. Thirdly, our retrospective study design limited our analysis, which is prone to bias due to unmeasured factors and hence cannot be used to infer causal relationships. Fourthly, certain drugs (e.g., corticosteroids) might cause hyperglycemia. This relationship was not assessed in our study, but we attempted to reduce it by utilizing just baseline blood glucose at admission. Fifthly, we were unable to analyze the relationship between hyperglycemia and long-term follow-up since we only collected hospitalization data. Lastly, we measured only one biomarker in a small population. There is a strong need for further research in a larger population examining other markers for DVT before femoral neck fracture surgery.

## Conclusions

In patients with femoral neck fractures, we found that hyperglycemia on admission has a dose–response association with preoperative DVT. Hyperglycemia has been associated with an increased incidence of DVT; these findings would provide vital information for thrombotic event prevention and preoperative optimization and might decrease death rates and complications during the perioperative time in femoral neck fracture patients. Indeed, additional new multicenter prospective randomized studies are needed to confirm our findings in the future.

## Supplementary Information

**Additional file1: eFigure 1.** Flow chart of enrollment. **eTable 1.** Multivariate Analysis for preoperative deep venous thrombosis. **eTable 2.** Patient Characteristics Before and After Propensity Score Matching by Glucose Level (≥6.10 mmol/L and <6.10mmol/L). **eTable 3.** Patient Characteristics Before and After Propensity Score Matching by Glucose Level (Group1 [0.00-5.30] vs Group2 [5.30-5.70] mmol/L). **eTable 4.** Patient Characteristics Before and After Propensity Score Matching by Glucose Level (Group2 [5.30-5.70] vs Group3 [5.70-6.60] mmol/L). **eTable 5.** Patient Characteristics Before and After Propensity Score Matching by Glucose Level (Group2 [5.30-5.70] vs Group4 [>6.60] mmol/L).

## Data Availability

All the data will be available upon motivated request to the corresponding author of the present paper.

## Abbreviations

DVT: Deep venous thrombosis
PTE: Pulmonary thromboembolism
VTE: Venous thromboembolism
IF: Internal fixation
CSS: Cannulated screw system
MI: Myocardial infarction
CT: Computed Tomography
MRI: Magnetic Resonance Imaging
ACCP: American College of Chest Physicians
LMWH: Low molecular weight heparin
NA: Not available
OR: Odds ratios
CI: Confidence intervals
SD: Standard deviation
