# Supplementary material for: Association between hyperglycemia on admission and preoperative deep venous thrombosis in patients with femoral neck fractures
Source: BMC Musculoskelet Disord. 2022 Oct 6;23:899. doi: 10.1186/s12891-022-05862-0 (PMC9535957; doi:10.1186/s12891-022-05862-0)
Supplement: Supplementary file 1 — Additional file1: eFigure 1. Flow chart of enrollment. eTable 1. Multivariate Analysis for preoperative deep venous thrombosis. eTable 2. Patient Characteristics Before and After Propensity Score Matching by Glucose Level (≥6.10 mmol/L and <6.10mmol/L). eTable 3. Patient Characteristics Before and After Propensity Score Matching by Glucose Level (Group1 [0.00-5.30] vs Group2 [5.30-5.70] mmol/L). eTable 4. Patient Characteristics Before and After Propensity Score Matching by Glucose Level (Group2 [5.30-5.70] vs Group3 [5.70-6.60] mmol/L). eTable 5. Patient Characteristics Before and After Propensity Score Matching by Glucose Level (Group2 [5.30-5.70] vs Group4 [>6.60] mmol/L). [file 12891_2022_5862_MOESM1_ESM.docx]

Appendix


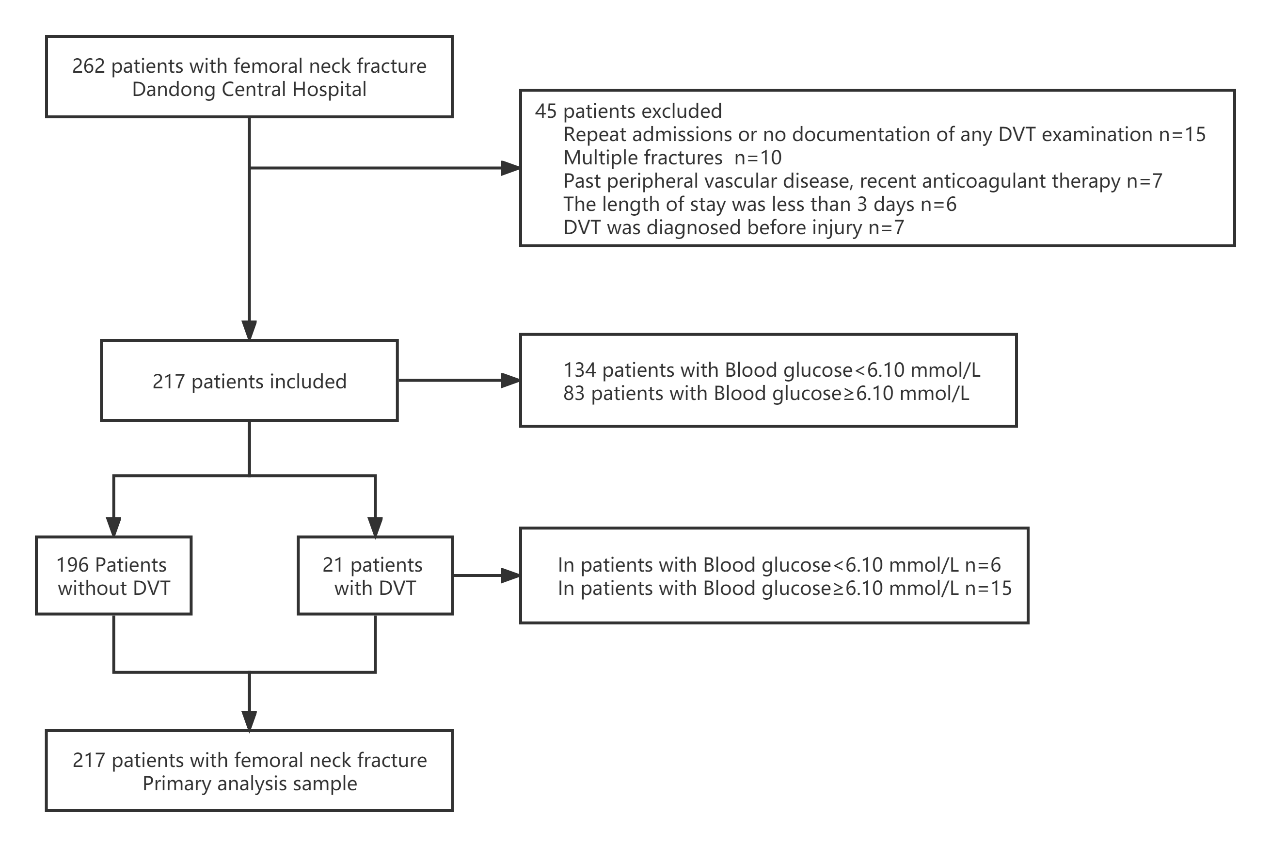


##### eFigure 1 Flow chart of enrollment

eTable 1 Multivariate Analysis for preoperative deep venous thrombosis

| Characteristics | Unadjusted | | Multivariable Regression Adjustment | |
| --- | --- | --- | --- | --- |
|  | OR (95% CI) | P | OR (95% CI) | P |
| Demographics |  |  |  |  |
| Mean age, years (SD) | 1.03(0.98-1.08) | 0.23 |  |  |
| Female gender, n (%) | 1.50(0.59-3.82) | 0.39 |  |  |
| Current Smoking, n (%) | 2.38(0.89-6.35) | 0.08 | 2.03(0.51-8.10) | 0.32 |
| Alcohol abuse, n (%) | 2.30(0.87-6.12) | 0.10 |  |  |
| Meantime from hospitalization to operation, days (SD) | 1.15(1.03-1.29) | 0.01 | 1.14(0.96-1.36) | 0.13 |
| Family history of VTE | 3.22(1.27-8.21) | 0.01 | 2.00(0.40-10.04) | 0.40 |
| Comorbidity, n (%) |  |  |  |  |
| Hypertension | 4.09(1.44-11.62) | 0.01 | 1.72(0.45-6.63) | 0.43 |
| Diabetes | 3.45(1.35-8.82) | 0.01 | 1.80(0.45-7.24) | 0.41 |
| Coronary artery disease (CAD) | 0.92(0.26-3.33) | 0.90 |  |  |
| Cerebrovascular Disease | 3.30(1.32-8.24) | 0.01 | 3.51(0.63-19.48) | 0.15 |
| Chronic Renal Failure | 2.17(0.57-8.26) | 0.26 |  |  |
| Chronic pulmonary disease | 1.55(0.42-5.76) | 0.51 |  |  |
| Malignancy | 0.84(0.10-6.86) | 0.87 |  |  |
| Time from injury to admission | 1.11(0.67-1.85) | 0.68 |  |  |
| Fractured limbs, n (%) | 1.19(0.49-2.94) | 0.70 |  |  |
| Baseline biomarker concentrations (Mean, SD) |  |  |  |  |
| White blood cell count, ×10^9/L | 1.06(0.88-1.27) | 0.55 |  |  |
| Neutrophil count，×10^9/L | 1.08(0.90-1.30) | 0.41 |  |  |
| Lymphocyte count，×10^9/L | 0.79(0.34-1.87) | 0.60 |  |  |
| Platelet count，×10^9/L | 1.00(0.99-1.00) | 0.35 |  |  |
| D-Dimer count，×ug/ml | 1.25(1.15-1.35) | <0.001 | 1.27(1.15-1.40) | <0.001 |
| Blood glucose, mmol/L | 4.71(1.75-12.68) | 0.002 | 3.03(0.77-11.87) | 0.11 |

##### eTable 2 Patient Characteristics Before and After Propensity Score Matching by Glucose Level (≥6.10 mmol/L and <6.10 mmol/L)

| Characteristics | Before matching | | | After matching | | |
| --- | --- | --- | --- | --- | --- | --- |
|  | Low Glu  (n=134) | High Glu  (n=83) | SMD | Low Glu  (n=57) | High Glu  (n=57) | SMD |
| Demographics |  |  |  |  |  |  |
| Mean age, years (SD) | 72.5(12.2) | 73.8(9.4) | 0.12 | 73.1(11.4) | 74.1(10.1) | 0.09 |
| Female gender | 93(69.4) | 59(71.1) | 0.04 | 38(66.7) | 39(68.4) | 0.04 |
| Current Smoking | 26(19.4) | 15(18.1) | 0.03 | 13(22.8) | 10(7.5) | 0.13 |
| Alcohol abuse | 23(17.2) | 19(22.9) | 0.14 | 10(17.5) | 14(24.6) | 0.17 |
| Meantime from hospitalization to operation, days (SD) | 5.1(2.9) | 6.1(3.6) | 0.32 | 6.1(3.4) | 5.8(3.1) | 0.08 |
| Family history of VTE | 23(17.2) | 23(27.7) | 0.25 | 15(26.3) | 13(22.8) | 0.08 |
| Comorbidity |  |  |  |  |  |  |
| Hypertension | 54(40.3) | 48(57.8) | 0.35 | 26(45.6) | 28(49.1) | 0.07 |
| Diabetes | 9(6.7) | 35(42.2) | 0.90 | 9(15.8) | 9(15.8) | <0.001 |
| Coronary artery disease (CAD) | 25(18.7) | 8(9.6) | 0.26 | 13(22.8) | 6(10.5) | 0.33 |
| Cerebrovascular disease | 33(24.6) | 27(32.5) | 0.18 | 16(28.1) | 17(29.8) | 0.04 |
| Chronic renal failure | 11(8.2) | 6(7.2) | 0.04 | 6(10.5) | 3(5.3) | 0.19 |
| Chronic pulmonary disease | 14(10.4) | 8(9.6) | 0.03 | 5(8.8) | 6(10.5) | 0.06 |
| Malignancy | 4(3.0) | 8(9.6) | 0.28 | 2(3.5) | 7(12.3) | 0.33 |
| Time from injury to admission |  |  |  |  |  |  |
| ≤12 h | 58(43.3) | 47(56.6) | 0.17 | 31(54.4) | 32(56.1) | 0.02 |
| 12–24 h | 33(24.6) | 11(13.3) |  | 9(15.8) | 6(10.5) |  |
| ≥24 h | 43(32.1) | 25(30.1) |  | 17(29.8) | 19(33.3) |  |
| Fractured limbs |  |  |  |  |  |  |
| Left | 73(54.5) | 39(47.0) | 0.15 | 35(61.4) | 29(50.9) | 0.21 |
| Right | 61(45.5) | 44(53.0) |  | 22(38.6) | 28(49.1) |  |
| Baseline biomarker concentrations（Mean, SD） |  |  |  |  |  |  |
| White blood cell count，×10^9/L | 8.0(2.3) | 9.1(2.3) | 0.51 | 8.0(2.1) | 9.0(2.1) | 0.47 |
| Neutrophil count，×10^9/L | 5.9(2.2) | 7.1(2.3) | 0.53 | 6.1(2.0) | 6.8(2.1) | 0.39 |
| Lymphocyte count，×10^9/L | 1.3(0.5) | 1.3(0.6) | 0.03 | 1.3(0.5) | 1.4(0.7) | 0.11 |
| Platelet count，×10^9/L | 208.1(68.5) | 216.6(61.9) | 0.13 | 202.8(76.3) | 218.8(64.6) | 0.23 |
| D-Dimer count，×ug/ml | 5.6(5.9) | 7.7(7.2) | 0.32 | 6.6(6.1) | 7.3(7.1) | 0.10 |

SMD: standardized mean difference; Glu: Glucose Level

##### eTable 3 Patient Characteristics Before and After Propensity Score Matching by Glucose Level (Group1 [0.00-5.30] vs Group2 [5.30-5.70] mmol/L)

| Characteristics | Before matching | | | After matching | | |
| --- | --- | --- | --- | --- | --- | --- |
|  | Group 1  (n=67) | Group 2  (n=49) | SMD | Group 1  (n=40) | Group 2  (n=40) | SMD |
| Demographics |  |  |  |  |  |  |
| Mean age, years (SD) | 72.2(12.5) | 72.8(12.7) | 0.05 | 71.8(11.7) | 72.2(13.0) | 0.03 |
| Female gender | 43(64.2) | 37(75.5) | 0.25 | 31(77.5) | 29(72.5) | 0.11 |
| Current Smoking | 16(23.9) | 6(12.2) | 0.30 | 3(7.5) | 6(15.0) | 0.24 |
| Alcohol abuse | 14(20.9) | 7(14.3) | 0.17 | 4(10.0) | 7(17.5) | 0.22 |
| Meantime from hospitalization to operation, days (SD) | 5.2(3.3) | 5.4(2.7) | 0.05 | 5.1(3.2) | 5.2(2.7) | 0.03 |
| Family history of VTE | 9(13.4) | 11(22.4) | 0.23 | 7(17.5) | 7(17.5) | <0.001 |
| Comorbidity |  |  |  |  |  |  |
| Hypertension | 23(34.3) | 21(42.9) | 0.17 | 16(40.0) | 17(42.5) | 0.05 |
| Diabetes | 5(7.5) | 2(4.1) | 0.14 | 1(2.5) | 2(5.0) | 0.13 |
| Coronary artery disease (CAD) | 14(20.9) | 10(20.4) | 0.01 | 7(17.5) | 8(20.0) | 0.06 |
| Cerebrovascular disease | 11(16.4) | 17(34.7) | 0.43 | 9(22.5) | 11(27.5) | 0.11 |
| Chronic renal failure | 7(10.4) | 3(6.1) | 0.16 | 4(10.0) | 3(7.5) | 0.09 |
| Chronic pulmonary disease | 10(14.9) | 3(6.1) | 0.29 | 5(12.5) | 2(5.0) | 0.26 |
| Malignancy | 2(3.0) | 1(2.0) | 0.06 | 1(2.5) | 0(0) | 0.22 |
| Time from injury to admission |  |  |  |  |  |  |
| ≤12 h | 23(34.3) | 28(57.1) | 0.51 | 14(35.0) | 23(57.5) | 0.56 |
| 12–24 h | 15(22.4) | 10(20.4) |  | 8(20.0) | 9(22.5) |  |
| ≥24 h | 29(43.3) | 11(22.4) |  | 18(45.0) | 8(20.0) |  |
| Fractured limbs |  |  |  |  |  |  |
| Left | 36(53.7) | 28(57.1) | 0.07 | 17(42.5) | 23(57.5) | 0.30 |
| Right | 31(46.3) | 21(42.9) |  | 23(57.5) | 17(42.5) |  |
| Baseline biomarker concentrations（Mean, SD） |  |  |  |  |  |  |
| White blood cell count，×10^9/L | 7.5(2.2) | 8.2(2.5) | 0.28 | 7.6(1.8) | 8.2(2.1) | 0.34 |
| Neutrophil count，×10^9/L | 5.4(2.0) | 6.1(2.4) | 0.30 | 5.5(1.6) | 6.1(2.1) | 0.31 |
| Lymphocyte count，×10^9/L | 1.4(0.6) | 1.3(0.5) | 0.29 | 1.4(0.6) | 1.3(0.5) | 0.17 |
| Platelet count，×10^9/L | 207.2(69.6) | 207.3(69.7) | 0.001 | 207.3(77.1) | 206.8(64.6) | 0.01 |
| D-Dimer count，×ug/ml | 4.9(5.8) | 7.0(6.2) | 0.35 | 5.9(6.8) | 5.8(5.4) | 0.01 |

SMD: standardized mean difference

##### eTable 4 Patient Characteristics Before and After Propensity Score Matching by Glucose Level (Group2 [5.30-5.70] vs Group3 [5.70-6.60] mmol/L)

| Characteristics | Before matching | | | After matching | | |
| --- | --- | --- | --- | --- | --- | --- |
|  | Group 2  (n=49) | Group 3  (n=47) | SMD | Group 2  (n=34) | Group 3  (n=34) | SMD |
| Demographics |  |  |  |  |  |  |
| Mean age, years (SD) | 72.8(12.7) | 74.5(10.0) | 0.15 | 71.9(13.6) | 74.2(9.8) | 0.19 |
| Female gender | 37(75.5) | 32(68.1) | 0.16 | 26(76.5) | 25(73.5) | 0.07 |
| Current Smoking | 6(12.2) | 9(19.1) | 0.19 | 4(11.8) | 4(11.8) | <0.001 |
| Alcohol abuse | 7(14.3) | 10(21.3) | 0.18 | 6(17.6) | 6(17.6) | <0.001 |
| Meantime from hospitalization to operation, days (SD) | 5.4(2.7) | 5.4(3.2) | 0.02 | 5.7(2.9) | 4.9(2.2) | 0.28 |
| Family history of VTE | 11(22.4) | 7(14.9) | 0.19 | 7(20.6) | 7(20.6) | <0.001 |
| Comorbidity |  |  |  |  |  |  |
| Hypertension | 21(42.9) | 23(48.9) | 0.12 | 15(44.1) | 14(41.2) | 0.06 |
| Diabetes | 2(4.1) | 5(10.6) | 0.25 | 2(5.9) | 2(5.9) | <0.001 |
| Coronary artery disease (CAD) | 10(20.4) | 4(8.5) | 0.34 | 8(23.5) | 2(5.9) | 0.51 |
| Cerebrovascular disease | 17(34.7) | 11(23.4) | 0.25 | 11(32.4) | 10(29.4) | 0.06 |
| Chronic renal failure | 3(6.1) | 3(6.4) | 0.01 | 3(8.8) | 3(8.8) | <0.001 |
| Chronic pulmonary disease | 3(6.1) | 6(12.8) | 0.23 | 3(8.8) | 4(11.8) | 0.10 |
| Malignancy | 1(2.0) | 5(10.6) | 0.36 | 1(2.9) | 4(11.8) | 0.34 |
| Time from injury to admission |  |  |  |  |  |  |
| ≤12 h | 28(57.1) | 24(51.1) | 0.11 | 20(58.8) | 18(52.9) | 0.24 |
| 12–24 h | 10(20.4) | 11(23.4) |  | 8(23.5) | 5(14.7) |  |
| ≥24 h | 11(22.4) | 12(25.5) |  | 6(17.6) | 11(32.4) |  |
| Fractured limbs |  |  |  |  |  |  |
| Left | 28(57.1) | 22(46.8) | 0.21 | 20(58.8) | 14(41.2) | 0.35 |
| Right | 21(42.9) | 25(53.2) |  | 14(41.2) | 20(58.8) |  |
| Baseline biomarker concentrations（Mean, SD） |  |  |  |  |  |  |
| White blood cell count，×10^9/L | 8.2(2.5) | 8.8(2.1) | 0.27 | 8.5(2.9) | 8.8(2.2) | 0.14 |
| Neutrophil count，×10^9/L | 6.1(2.4) | 6.6(2.3) | 0.22 | 6.4(2.7) | 6.8(2.0) | 0.19 |
| Lymphocyte count，×10^9/L | 1.3(0.5) | 1.3(0.4) | 0.02 | 1.2(0.5) | 1.3(0.4) | 0.12 |
| Platelet count，×10^9/L | 207.3(69.7) | 226.3(65.4) | 0.28 | 198.6(61.4) | 241.6(67.0) | 0.67 |
| D-Dimer count，×ug/ml | 7.0(6.2) | 6.4(6.6) | 0.09 | 7.4(6.4) | 6.4(6.9) | 0.14 |

SMD: standardized mean difference

##### eTable 5 Patient Characteristics Before and After Propensity Score Matching by Glucose Level (Group2 [5.30-5.70] vs Group4 [>6.60] mmol/L)

| Characteristics | Before matching | | | After matching | | |
| --- | --- | --- | --- | --- | --- | --- |
|  | Group 2  (n=49) | Group 4  (n=54) | SMD | Group 2  (n=22) | Group 4  (n=22) | SMD |
| Demographics |  |  |  |  |  |  |
| Mean age, years (SD) | 72.8(12.7) | 73.0(9.1) | 0.02 | 72.7(10.9) | 72.2(10.2) | 0.05 |
| Female gender | 37(75.5) | 40(74.1) | 0.03 | 16(72.7) | 14(63.6) | 0.19 |
| Current Smoking | 6(12.2) | 10(18.5) | 0.17 | 6(27.3) | 5(22.7) | 0.10 |
| Alcohol abuse | 7(14.3) | 11(20.4) | 0.16 | 4(18.2) | 6(27.3) | 0.21 |
| Meantime from hospitalization to operation, days (SD) | 5.4(2.7) | 6.1(3.6) | 0.25 | 5.7(2.8) | 6.3(3.7) | 0.18 |
| Family history of VTE | 11(22.4) | 19(35.2) | 0.28 | 5(22.7) | 6(27.3) | 0.10 |
| Comorbidity |  |  |  |  |  |  |
| Hypertension | 21(42.9) | 35(64.8) | 0.45 | 11(50.0) | 11(50.0) | <0.001 |
| Diabetes | 2(4.1) | 32(59.3) | 1.46 | 2(9.1) | 2(9.1) | <0.001 |
| Coronary artery disease (CAD) | 10(20.4) | 5(9.3) | 0.31 | 5(22.7) | 2(9.1) | 0.37 |
| Cerebrovascular disease | 17(34.7) | 21(38.9) | 0.09 | 9(40.9) | 9(40.9) | <0.001 |
| Chronic renal failure | 3(6.1) | 4(7.4) | 0.05 | 2(9.1) | 1(4.5) | 0.18 |
| Chronic pulmonary disease | 3(6.1) | 3(5.6) | 0.02 | 2(9.1) | 1(4.5) | 0.18 |
| Malignancy | 1(2.0) | 4(7.4) | 0.25 | 1(4.5) | 4(18.2) | 0.43 |
| Time from injury to admission |  |  |  |  |  |  |
| ≤12 h | 28(57.1) | 30(55.6) | 0.10 | 14(63.6) | 14(63.6) | 0.06 |
| 12–24 h | 10(20.4) | 8(14.8) |  | 4(18.2) | 3(13.6) |  |
| ≥24 h | 11(22.4) | 16(29.6) |  | 4(18.2) | 5(22.7) |  |
| Fractured limbs |  |  |  |  |  |  |
| Left | 28(57.1) | 26(48.1) | 0.18 | 11(50.0) | 11(50.0) | <0.001 |
| Right | 21(42.9) | 28(51.9) |  | 11(50.0) | 11(50.0) |  |
| Baseline biomarker concentrations（Mean, SD） |  |  |  |  |  |  |
| White blood cell count，×10^9/L | 8.2(2.5) | 9.4(2.4) | 0.47 | 7.7(1.6) | 9.5(2.5) | 0.82 |
| Neutrophil count，×10^9/L | 6.1(2.4) | 7.3(2.3) | 0.49 | 5.7(1.9) | 7.1(2.2) | 0.71 |
| Lymphocyte count，×10^9/L | 1.3(0.5) | 1.4(0.7) | 0.21 | 1.2(0.5) | 1.6(1.0) | 0.47 |
| Platelet count，×10^9/L | 207.3(69.7) | 207.1(58.0) | 0.003 | 213.1(71.2) | 209.4(60.1) | 0.06 |
| D-Dimer count，×ug/ml | 7.0(6.2) | 7.7(7.2) | 0.11 | 6.2(6.5) | 6.1(6.6) | 0.02 |

SMD: standardized mean difference
